# Chronic Compartment Syndrome in Athletes

**DOI:** 10.1055/s-0044-1787766

**Published:** 2024-07-15

**Authors:** Pedro Baches Jorge, Mariana Belaunde Toledo, Flora Chaves Mari, Rodrigo Ruas Floriano de Toledo, Marcos Vaz de Lima, Jan Willem Cerf Sprey

**Affiliations:** 1 Grupo de Trauma do Esporte, Departamento de Ortopedia e Traumatologia, Irmandade de Misericórdia da Santa Casa de São Paulo, São Paulo, SP, Brasil; 2 Grupo de Medicina do Esporte, Departamento de Ortopedia e Traumatologia, Irmandade de Misericórdia da Santa Casa de São Paulo, São Paulo, SP, Brasil

**Keywords:** athletes, compartment syndromes, sports injuries, sports medicine

## Abstract

Chronic compartment syndrome (CCS) is a pressure increase within a non-expandable fibro-osseous space resulting from continuous and intense physical activity. Its symptoms usually improve with rest or reduced activity. It is a critical cause of lower limb pain in athletes and the second most common cause of effort-related leg pain. Less frequent reports include CCS in the lumbar paravertebral compartments, in the hand, the forearm, the thigh, and the foot. Although CCS mainly affects long-distance runners, it may also occur in sports such as lacrosse, football, basketball, skiing, and field hockey. Muscle tension, cramps, symptoms worsening with physical exercise, pain, and reduced sensitivity in the upper part of the foot are the main CCS findings, and diagnosis is essentially clinical. Even though controversial and with some limitations, CCS diagnosis has relied on measuring the intracompartmental pressure after exercise. However, new alternative tools are under study, particularly those less invasive, such as magnetic resonance imaging (MRI) after the exercise protocol. For years, open fasciotomy was the most relevant treatment for CCS in athletes, but new surgical techniques are gaining importance, such as minimally-invasive fasciotomy and endoscopic procedures. Some conservative therapies hold promise as potential alternatives for patients who do not want surgery, but robust evidence to support them remains lacking, especially for athletes.

## Introduction

Von Volkmann first described compartment syndrome (CS) in 1881. Subsequently, there were CS reports in several anatomical regions, especially the lower extremities. 1 In addition, although there are rarer and limited CS cases reported in the hand, forearm, and paravertebral compartments. 1 2 3

Compartment syndrome is an increased pressure within a non-expandable fibro-osseous space, leading to compromised tissue perfusion in that specific area. Reduced perfusion initially causes ischemic pain, followed by reversible and eventually irreversible tissue damage within the compartment. The resulting edema creates a vicious cycle, further aggravating the ischemic injury. 1

Compartment syndrome manifestation can be acute, characterized by severe symptoms over a short period, or chronic, lasting for a long time. Acute compartment syndrome (ACS) is a medical emergency, typically resulting from a severe injury or trauma that leads to intense pain. On the other hand, chronic CS (CCS) is often not an immediate medical emergency, and it appears after intense and repetitive athletic activity in the absence of acute trauma. Its treatment involves rest and recovery. 4 5

Along with other exertion-related conditions, CCS can significantly contribute to developing exertion-dependent symptoms. Therefore, this condition has particular importance in sports and physical exercise. One of the first descriptions of CCS occurred during the British expedition to the South Pole in 1912, in which Edward Wilson described swelling and pain in the anterior compartment of the leg during long walks in the Antarctic region. Subsequent historical records have also emphasized the prevalence of CCS in military cohorts, leading to the nickname “marching gangrene”. 4

## Pathophysiology

Numerous hypotheses have attempted to elucidate CCS pathophysiology. However, the true etiology and development process of the condition remain undetermined. 6

We know that intense exercise can lead to substantial increases in muscle volume resulting from increased metabolic demands, tissue perfusion, and muscle fiber growth. Compartment syndrome occurs when the pressure within the fascial compartment exceeds the diastolic pressure. This compromised blood flow causes tissue ischemia, metabolite accumulation, and pain in the affected area. 4

Some researchers have suggested that untreated CCS can result in neural compression and irreversible damage due to fluid leakage and increased intracompartmental pressures. Decreased capillary density and impaired venous flow have also been implicated in CCS development. 4

Chronic CS results from increased intracompartmental pressures potentially triggered by several precipitating events. Local blood flow, determined by local arterial pressure, venous pressure, and vascular resistance, impacts pressure within a muscular compartment. Ischemia occurs when interstitial pressure exceeds capillary perfusion pressure (CPP). Skeletal muscle ischemia releases a histamine-like substance that increases vascular permeability, causing the formation of blood thrombi and worsening ischemic conditions. Myocyte rupture releases proteins, which results in water escaping from the arterial blood into the compartment. 7

Other contributing factors associated with CCS involve inadequate training methods, limb misalignment, leg length discrepancies, running style, and poor neuromuscular control. 8

Although the precise underlying mechanism is still debatable, consensus points to CCS development resulting from muscular effort repetition within a compartment, which reduces blood perfusion. 8

## Epidemiology and Risk Factors

The precise prevalence of CCS remains uncertain due to factors that include self-treatment or activity modification, errors in clinical diagnosis, and failure to seek medical care. It is estimated that 14 to 34% of leg pain referred for orthopedic treatment because of activity or exertion is consistent with CCS. 4 The current prevalence of CCS in the general population is unknown. However, it has been documented in specific athletic subgroups at a rate of 0.49 cases per one thousand people per year. 9

Chronic compartment syndrome primarily affects the leg, with more than 95% of cases reported in this region. However, there are variable reports of involvement in the lumbar paravertebral compartment, 1 hand, forearm, thigh, and foot from specific high-risk groups. 4 Among leg compartments, the most affected is the anterior compartment (42–60%), followed by the lateral (35–36%), deep posterior (19–32%), and superficial posterior compartments (3–21%). Single-compartment involvement is less common (37%); approximately 40% of symptomatic cases involve 2 compartments, 18% involve 3 compartments, and only 5% affect all 4 compartments. Bilateral involvement is more prevalent, representing up to 95% of cases, with no differences in laterality. 4

Anatomical disparities between adolescents and adults increase the risk of CS development in younger patients. 7 Shadgan et al. 10 noted clinicians often believe that younger subjects have a stiffer/narrower and stronger fascia, combined with greater muscle density, increasing their vulnerability to CS. Therefore, CCS diagnosis is frequent, although not limited to, in young athletes involved in repetitive activities, such as long-distance or cross-country running. 7

Older studies showed conflicting outcomes regarding the prevalence of CCS in men and women. Some studies reported a higher CCS occurrence in men, while others have suggested a potential increased occurrence in women. However, the latest literature investigations reported a similar incidence of CSS among men and women. 4

Rothman et al. 11 found that women were less likely than men to return to sports after a surgical intervention, and factors such as intracompartmental pressures, sports participation, and postoperative outcomes were not statistically different between genders.

More than 90% of people diagnosed with CSS participate in athletic activities, with no difference reported between those participating in elite or recreational levels of competition. Although several sports have been linked to CCS, including lacrosse, football, basketball, skiing, and field hockey, the condition is more frequent in endurance runners, who account for up to 68% of cases. 4 Some rare cases have occurred in weightlifting, American football, and baseball athletes. 2 3 12 However, CCS can also affect less active populations.

Intense exercise, including running, although not exclusively, has been associated with an increased CCS incidence. Physiological and metabolic changes due to significant physical activity affect muscle volume and compartmental pressures. Eccentric muscle strengthening in adults is a potential cause for decreased fascial conformity and CCS development. Patients with CCS often have a thickened fascia and a higher prevalence of fascial defects compared with asymptomatic individuals. Anabolic androgenic steroids and other performance-enhancing drugs for muscle growth may also contribute to abnormal elevations in intracompartmental pressures, and some suggested them as potential risk factors for CCS. 4 Some evidence indicated that training errors, specifically an abrupt increase in training volume, intensity, or both, can be the chief risk factor for CCS development. 13

## Anatomy and Clinical Presentation

Chronic CS is the second most common cause of exertion-related leg pain, followed by medial tibial stress syndrome (MTSS), affecting approximately one third of athletes. It is critical to consider other potential diagnoses of exercise-induced leg pain, such as nerve entrapment, bone stress injuries, deep vein thrombosis, MTSS, and miscellaneous clinical conditions during symptom evalution. 4 13

**Fig. 1:**
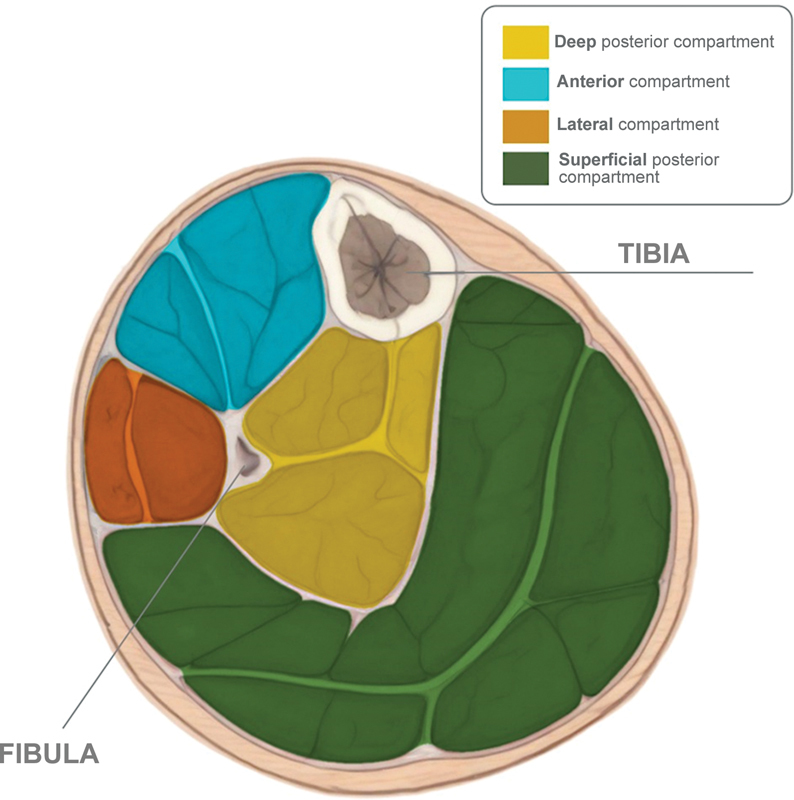
A transverse section of the leg shows the four compartments, namely, the anterior compartment (containing the tibialis anterior, extensor digitorum longus, extensor hallucis longus, and fibularis tertius muscles), the lateral compartment (composed of the peroneus longus and brevis muscles), the superficial posterior compartment (containing the gastrocnemius, soleus, and plantaris muscles), and the deep posterior compartment (containing the tibialis posterior and the flexor digitorum longus muscles).

A comprehensive knowledge of lower limb anatomy is crucial for CCS diagnosis and identification of the compartments involved. The lower leg anatomically has four compartments, namely, anterior, lateral, superficial posterior, and deep posterior, with an additional fifth compartment for the tibialis posterior muscle, which has a fascia ( Fig. 1 ). 14

The anterior compartment contains the deep peroneal nerve, anterior tibial artery, anterior tibialis muscle, extensor digitorum longus muscle, extensor hallucis longus muscle, and fibularis tertius muscle. Increased pressure in this compartment can lead to sensory loss in the first interdigital space and weakness during toe and ankle dorsiflexion. 14

The lateral compartment has the peroneus longus and brevis muscles, the peroneal artery, and the superficial peroneal nerve. Compression in this compartment can result in weakness during foot eversion and reduced sensation in the dorsum of the foot. 14

The posterior superficial compartment contains the posterior tibial artery, gastrocnemius, soleus, and plantaris muscles, in addition to the distal segment of the sural nerve. Its compression can cause numbness on the side of the foot and distal calf. 14

The deep posterior compartment has the tibialis posterior muscle, flexor digitorum longus muscles, peroneal artery, and tibial nerve. Increased pressure in this compartment can lead to weakness in plantar flexion and numbness in the sole. 14 Since most cases occur in the lower limbs, we will not discuss anatomical details from other regions.

The natural progression of CCS is often atraumatic, although some subjects may report a history of low-energy trauma. Patients usually experience stiffness, pain, or discomfort in the anterior and lateral part of the leg after prolonged exercise, and symptoms frequently improve with rest or activity reduction; this particular detail is a specific criterion for the disease. 13 The symptoms of CCS are bilateral in up to 95% of patients. At the superficial peroneal nerve distribution, affected subjects may experience reduced vibratory sensitivity and decreased motor range, leading to lower foot and ankle control loss described before. 4 Cramps, hyposensitivity, or muscle weakness are evident in approximately one third of patients. 13

## Diagnosis

The diagnosis of CCS relies on a detailed history and thorough physical examination. It is vital to document training frequency, volume, duration, and intensity, along with any patterns for the onset and resolution of reported symptoms. Although patients may not experience symptoms at rest, exertion can trigger significant symptoms that limit activity. The top five symptoms frequently reported by patients with CCS include pain, tightness, cramping, weakness, and decreased sensation in the dorsum of the foot. 4

Recently, Vogels et al. 15 proposed five key criteria for CCS diagnosis. Study panel members agreed that CCS was likely if the patient (I) participates in activities requiring repetitive activation of the same muscles, (II) reports pain during exercise, (III) reports stiffness/tightness during exercise, (IV) stops specific activities earlier or avoids them, and (V) presents symptoms induced by provocative activities at the physical examination. 15

During the physical examination, passive compartment stretching may elicit pain if the patient has recently exercised, although pain is uncommon at rest. Palpation of the affected area can reveal fascial defects in 39 to 46% of subjects with CCS. 4

Although there is much ongoing debate, the historical standard for diagnosing CCS has been intracompartmental pressure measurement. Whitesides et al. 16 pioneered the development of a technique for this measurement in a revolutionary study using objects such as a syringe, needle, saline solution, and plastic tubes connected to a manometer. Current intracompartmental pressure monitoring can employ several commercially available devices inspired by Whitesides' invention. 16 This monitoring allows a comparison between affected and unaffected compartments in both lower limbs. In the diagnostic process, patients undergo a physical stress test before and after a series of manometry measurements to analyze trends in intracompartmental pressures in symptomatic compartments. Typical resting intracompartmental pressure in the leg is usually lower than 10 mmHg, although measurements can vary considerably between patients and suffer influence by the operator performing the procedure. 4

A study by Davis et al. 17 monitored 17 patients with CCS during physical stress tests. Analysis revealed that these subjects experienced leg pain after, on average, 11 minutes of exertion, rating the pain as an 8 out of 10 on the Visual Analog Scale. Symptoms subsided after about 45 minutes of rest. Approximately 36% of subjects reported numbness or tingling in addition to pain after exertion. Objective pressure measurements showed significant increases in the anterior, lateral, deep posterior, and superficial posterior compartments following physical stress testing. 4

Pedowitz et al. 18 established diagnostic criteria to confirm exercise-induced CCS. According to them, the diagnosis requires meeting the following criteria: 1) preexercise pressure higher than 15 mmHg; (2) pressure 1 minute after exercise higher than 30 mmHg; or 3) pressure 5 minutes after exercise higher than 20 mmHg.

However, it is worth highlighting that diagnostic cut-off criteria differ substantially depending on the author, 15 and pressure measurements are not always reliable due to factors such as patient tolerance, operator technique, and use of different measuring devices. Furthermore, the invasive nature of the test may be associated with risks of incorrect needle placement, bruising, and nerve damage. 15

Aweid et al. 4 reviewed several studies evaluating the usefulness of intracompartmental pressure measurements for CCS diagnosis. These authors concluded that although pressure measurement use is widely available, there is limited evidence to validate their accuracy, and the clinical presentation should be further considered for CCS diagnosis.

Regarding the applicability and diagnostic value of measuring intracompartmental pressure, it is essential to remember that the clinical history, physical examination, and exclusion of differential diagnoses are indispensable to the diagnostic process. To overcome these limitations of the needle technique, new diagnostic protocols have been suggested, with the recommended systematic use of conventional MRI to exclude differential diagnoses. 19

Magnetic resonance imaging is the best imaging method for evaluating exercise-related leg pain, as it detects conditions such as tibial stress syndrome, tibial stress fracture, neural compressions, muscle and tendon injuries, exercise-related thrombosis, and fascial hernias. 19

In clinical practice, conventional MRI sequences initially exclude differential diagnoses. Subsequently, patients run (or walk) on a treadmill, according to their physical capabilities, until they can no longer tolerate the activity due to pain. Immediately after stopping the activity, patients undergo a new MRI for fluid-sensitive and fat-suppressed axial sequence (T2-weighted/short tau inversion recovery, STIR) acquisition. Some studies have confirmed the validity of postexercise MRI for CCS diagnosis, using a 1.54-fold increase in signal intensity as a diagnostic cut-off value with a 96% sensitivity and 87 to 90% specificity. 19

Therefore, MRI is a non-invasive method, readily accepted by patients, with good availability in large medical centers, the best imaging test to rule out differential diagnoses, and a scientifically validated option for CCS diagnosis. 19

## Treatment

### Surgical Treatment

The treatment for CCS comprises several surgical and non-operative management strategies. Traditionally, surgical treatment has more reports and better outcomes, but there is increasing evidence that conservative treatment may be an option in selected cases. In athletes, management consists of surgical intervention. Non-surgical treatment failure, paresthesia, exertion-induced pain that disappears with rest, tightness, cramps, ischemia, foot drop, and the patient's desire are the main indications for surgical treatment. 9 20

In a systematic review of the surgical management of CCS 9 including 1,495 patients from 24 studies, the most used techniques were compartment-specific open fasciotomy (86%), fasciotomy with partial fasciectomy (12%), and endoscopic fasciotomy (< 2%). For the anterior compartment, the most commonly affected by CSS, a single longitudinal incision between the anterior tibial crest and fibula through the skin and subcutaneous tissue was the most frequent procedure (207 out of 240). However, the outcomes of this review did not demonstrate a superiority between the techniques described. Concerning the posterior compartment, the most used surgical technique was a longitudinal incision slightly medial to the tibial crest with the release of the solar bridge of the tibia to approach the deep fascia. The success rate of this intervention was 61% for the deep posterior compartment (44 out of 72) and 100% for the superficial posterior (3 out of 3). The authors suggested that this compartment is prone to lower surgical success.

A systematic review including seven articles on surgical intervention in the posterior compartment found that the techniques differed slightly throughout the studies. However, the review reached no conclusions since the researchers used different methods for outcome measurement. 21

New procedures, such as minimally-invasive or endoscopic procedures, have been gaining relevance in recent decades. A review by Lohrer et al. 13 concluded the lack of statistical difference between these techniques since the unweighted average success rate was 86.3% for the endoscopic technique and 80.0% for the minimally invasive CCS release. D'Amore et al. 22 compared endoscopic procedures and open fasciotomy in elite and amateur athletes with lower limb CCS. Their results showed that the return to sport rate was 84.6% in patients undergoing an endoscopic procedure and 72.7% in those undergoing open fasciotomy, with symptom recurrence rates of 69.2% and 72.7%, respectively, with no statistical difference. Neither group presented complications or severe outcomes.

The endoscopic technique would have benefits over open fasciotomy, such as lower risk of infection, shorter time to activity return due to the lower soft-tissue manipulation, less postoperative hematoma, limited fibrosis, better visualization of compartmental structures, and fascial release extension. 9 22 It is an adequate alternative treatment for CCS release from the anterior and lateral compartments, with a good success rate and no inferiority in the literature. 22

## Conservative Treatment

Exercise-induced CCS conservative treatment remains poorly documented in scientific research. Rest, interrupting symptom-triggering activity, and analgesic agents seem essential. However, few documented guidelines or specific procedures describe how to optimize them and the population most benefited from those interventions. In a systematic review of the literature on new non-surgical management, Rajasekaran et al. 6 found little evidence of techniques, which included gait shifting, chemodenervation, ultrasound-guided fascia fenestration, and massage.

Nevertheless, a case series by Diebal et al. 23 on different running techniques and how they affect compartment pressure and pain in CCS patients showed promise, and it was included in a military non-surgical management program. 23 24 This protocol involves several treatments described in the literature, with a greater focus on walking and running re-education. In one study, these authors reported that, after a 2-year follow-up in a population of 50 patients undergoing their protocol, 57% were still on active duty without surgery, 43% returned to their original military post, 36% left military service, 48% remained with symptoms, and 12% of patients underwent fasciotomy. 24 This study showed moderate outcomes, which could decrease the need for surgical procedures. Although there is little evidence on the results of gait retraining in athletes, this research's focus may be an alternative to fasciotomy or an attempt to prevent it.

In 2022, a clinical consensus panel of experts discussed conservative CCS treatment and its efficacy. It concluded that gait retraining and cessation of provocative activities are critical when attempting a non-surgical approach. The literature cites physical therapy, botulinum injections, and shoe modifications as less significant and mainly adjacent measures depending on the patient's symptoms. 15

## Conservative versus Surgical Treatment

Although there are no randomized clinical trials to compare surgical and conservative treatments, some studies reported a superiority of interventional procedures, mainly in patients with CCS of the anterior compartment and amateur and elite athletes. In a retrospective cohort, Vogels et al. 25 found that the success rate considered by patients was significantly higher in those who underwent fasciotomy (42% compared with a 17% success rate in the group undergoing conservative treatment), and a lower frequency of pain and tightness during sports was also noted. However, there was no difference between these two groups regarding the return to the same level of performance before each intervention.

In contrast to these findings, Thein et al. 26 observed significantly better outcomes in returning to sports and maintaining the same physical activity level per the Tegner score in patients undergoing surgical treatment. The rate of return to presymptomatic athletic level was 25% in patients who did not undergo fasciotomy and 77.4% for those who did, with *p* = 0.001.

It is worth highlighting that none of these studies have the gold standard regarding the methodological procedure, with several inherent biases in their conduction, and no standardized conservative treatment, which was mostly performed by physical therapists or clinicians. However, they corroborate the hypothesis that the surgical procedure seems to be a better treatment, with higher patient satisfaction, in athletes. 25 26

## Final Considerations

Chronic CS is the second most common cause of exertion-induced lower limb pain, followed by MTSS. Although it is rarely urgent and symptoms are relieved by rest and cessation of triggering activities, accurate assessment of intracompartmental pressure remains challenging, and gold-standard treatment is imprecise. Furthermore, the authors suggest that untreated CCS can lead to neural compression and irreversible damage due to elevated intracompartmental pressures.

The true prevalence of CCS remains uncertain, but it is estimated to account for approximately 14 to 34% of physical activity-related leg pain. Intense exercise, particularly running, has been linked to an increased incidence of CCS. However, military personnel are also usually affected by CCS.

## References

[1] Alexander W Low N Pratt G Acute lumbar paraspinal compartment syndrome: a systematic review ANZ J Surg 2018 88 09 854 859 29316189 10.1111/ans.14342

[2] Mattiassich G Larcher L Leitinger M Trinka E Wechselberger G Schubert H Paravertebral compartment syndrome after training causing severe back pain in an amateur rugby player: report of a rare case and review of the literature BMC Musculoskelet Disord 2013 14 259 24004522 10.1186/1471-2474-14-259 PMC3848892

[3] Willick S E Deluigi A J Taskaynatan M Petron D J Coleman D Bilateral chronic exertional compartment syndrome of the forearm: a case report and review of the literature Curr Sports Med Rep 2013 12 03 170 174 23669087 10.1249/JSR.0b013e3182913c82

[4] Dunn J C Waterman B R Chronic exertional compartment syndrome of the leg in the military Clin Sports Med 2014 33 04 693 705 25280617 10.1016/j.csm.2014.06.010

[5] American Academy of Orthopaedic Surgeons.Compartment SyndromeAvailable from: https://orthoinfo.aaos.org/en/diseases–conditions/compartment-syndrome/

[6] Rajasekaran S Hall M M Nonoperative Management of Chronic Exertional Compartment Syndrome: A Systematic Review Curr Sports Med Rep 2016 15 03 191 198 27172084 10.1249/JSR.0000000000000261

[7] McLaughlin N Heard H Kelham S Acute and chronic compartment syndromes: know when to act fast JAAPA 2014 27 06 23 26 10.1097/01.JAA.0000446999.10176.13 24819953

[8] Buerba R A Fretes N F Devana S K Beck J J Chronic exertional compartment syndrome: current management strategies Open Access J Sports Med 2019 10 10 71 79 31213933 10.2147/OAJSM.S168368 PMC6537460

[9] Campano D Robaina J A Kusnezov N Dunn J C Waterman B R Surgical Management for Chronic Exertional Compartment Syndrome of the Leg: A Systematic Review of the Literature Arthroscopy 2016 32 07 1478 1486 27020462 10.1016/j.arthro.2016.01.069

[10] Shadgan B Menon M Sanders D Current thinking about acute compartment syndrome of the lower extremity Can J Surg 2010 53 05 329 334 20858378 PMC2947124

[11] Rothman R Berke C Jivanelli B Casey E Cheng J Sex and gender differences in lower limb chronic exertional compartment syndrome: a systematic review[published online ahead of print, 2023 Feb 7]Phys Sportsmed 2024 52 01 1 11 36698053 10.1080/00913847.2023.2173489

[12] Hiramatsu K Yonetani Y Kinugasa K Deep peroneal nerve palsy with isolated lateral compartment syndrome secondary to peroneus longus tear: a report of two cases and a review of the literature J Orthop Traumatol 2016 17 02 181 185 26362782 10.1007/s10195-015-0373-8 PMC4882295

[13] Lohrer H Nauck T Lohrer L Endoscopic-assisted Release of Lower Leg Chronic Exertional Compartment Syndromes: Results of a Systematic Literature Review Sports Med Arthrosc Rev 2016 24 01 19 23 26752774 10.1097/JSA.0000000000000106

[14] Tucker A K Chronic exertional compartment syndrome of the leg Curr Rev Musculoskelet Med 2010 3(1-4):32 37 21063498 10.1007/s12178-010-9065-4 PMC2941579

[15] Vogels S Ritchie E D van der Burg B LSB Scheltinga M RM Zimmermann W O Hoencamp R Clinical Consensus on Diagnosis and Treatment of Patients with Chronic Exertional Compartment Syndrome of the Leg: A Delphi Analysis Sports Med 2022 52 12 3055 3064 35904751 10.1007/s40279-022-01729-5 PMC9691483

[16] Whitesides T E Jr Haney T C Harada H Holmes H E Morimoto K A simple method for tissue pressure determination Arch Surg 1975 110 11 1311 1313 1191023 10.1001/archsurg.1975.01360170051006

[17] Davis D E Raikin S Garras D N Vitanzo P Labrador H Espandar R Characteristics of patients with chronic exertional compartment syndrome Foot Ankle Int 2013 34 10 1349 1354 23669162 10.1177/1071100713490919

[18] Pedowitz R A Hargens A R Mubarak S J Gershuni D H Modified criteria for the objective diagnosis of chronic compartment syndrome of the leg Am J Sports Med 1990 18 01 35 40 2301689 10.1177/036354659001800106

[19] Nico M AC Carneiro B C Zorzenoni F O Ormond Filho A G Guimarães J B The Role of Magnetic Resonance in the Diagnosis of Chronic Exertional Compartment Syndrome Rev Bras Ortop 2020 55 06 673 680 10.1055/s-0040-1702961 PMC7748934 33364643

[20] Vajapey S Miller T L Evaluation, diagnosis, and treatment of chronic exertional compartment syndrome: a review of current literature Phys Sportsmed 2017 45 04 391 398 28952402 10.1080/00913847.2017.1384289

[21] Winkes M B Hoogeveen A R Scheltinga M R Is surgery effective for deep posterior compartment syndrome of the leg? A systematic review J Vasc Surg 2014 59 06 1677 1686 24065078 10.1136/bjsports-2013-092518

[22] D'Amore T Rao S Gawel R J Return to Sport Rates and Subjective Outcomes Are Similar After Open or Endoscopically Assisted Compartment Release for Chronic Lower-Extremity Exertional Compartment Syndrome Arthrosc Sports Med Rehabil 2022 4 06 e1953 e1959 36579030 10.1016/j.asmr.2022.08.003 PMC9791877

[23] Diebal A R Gregory R Alitz C Gerber J P Forefoot running improves pain and disability associated with chronic exertional compartment syndrome Am J Sports Med 2012 40 05 1060 1067 22427621 10.1177/0363546512439182

[24] Zimmermann W O Hutchinson M R Van den Berg R Hoencamp R Backx F JG Bakker E WP Conservative treatment of anterior chronic exertional compartment syndrome in the military, with a mid-term follow-up BMJ Open Sport Exerc Med 2019 5 01 e000532 10.1136/bmjsem-2019-000532 PMC6539157 31191976

[25] Vogels S Ritchie E D Hundscheid H P Chronic Exertional Compartment Syndrome in the Leg: Comparing Surgery to Conservative Therapy Int J Sports Med 2021 42 06 559 565 33176383 10.1055/a-1273-7777

[26] Thein R Tilbor I Rom E Return to sports after chronic anterior exertional compartment syndrome of the leg: Conservative treatment versus surgery J Orthop Surg (Hong Kong) 2019 27 02 2.309499019835651E15 10.1177/2309499019835651 30909799

