# Supplementary material for: Chronic Compartment Syndrome in Athletes
Source: Rev Bras Ortop (Sao Paulo). 2024 Jul 15;59(5):e650–6. doi: 10.1055/s-0044-1787766 (PMC11624925; doi:10.1055/s-0044-1787766)
Supplement: Related texts

# Research article: A síndrome compartimental crônica em atletas

**Keywords:** atletas, medicina do esporte, sindromes compartimentais, traumatismos em atletas

## Abstract

A síndrome compartimental crônica (SCC) pode ser definida como uma elevação da pressão dentro de um espaço fibro-ósseo não expansível, que surge por meio de atividades físicas contínuas e intensas, com sintomas que geralmente melhoram com repouso ou redução da atividade. É uma causa importante de dor nos membros inferiores em atletas, sendo a segunda causa mais comum de dores nas pernas relacionada ao esforço, embora existam relatos menos comuns em compartimentos paravertebrais lombares, na mão, no antebraço, na coxa e no pé. É mais frequentemente observada em corredores de longa distância; porém, outros esportes também têm sido associados a essa entidade, como lacrosse, futebol, basquete, esqui e hóquei de campo. A tensão muscular, cãibras, piora dos sintomas com o exercício físico, dor e redução da sensibilidade na parte superior do pé são os principais sintomas relacionados a essa síndrome, sendo o diagnóstico essencialmente clínico. Embora controversa e com algumas limitações, a medição da pressão intracompartimental após o exercício tem sido utilizada para o diagnóstico da SCC. No entanto, novas ferramentas estão sendo estudadas como alternativa, especialmente aquelas que são menos invasivas, como a imagem de ressonância magnética (RM) após protocolo de exercício. A fasciotomia aberta foi por

vários anos o tratamento mais relevante para SCC em atletas, porém novas técnicas cirúrgicas estão ganhando importância, como a fasciotomia minimamente invasiva e o procedimento endoscópico. Alguns tratamentos conservadores são promissores como alternativas possíveis para aqueles que não desejam a cirurgia, mas ainda há falta de evidências robustas para sustentá-los, especialmente para atletas.

## Introdução

A síndrome compartimental (SC) foi descrita inicialmente por von Volkmann em 1881. Posteriormente, a SC foi relatada em várias regiões anatômicas, com as extremidades inferiores sendo mais comumente afetadas, 1 embora haja casos mais raros e limitados relatados na mão, no antebraço e no compartimento paravertebral. 1 2 3

A SC pode ser definida como a elevação da pressão dentro de um espaço fibro-ósseo não expansível, levando a comprometimento da perfusão tecidual naquela área específica. A redução da perfusão inicialmente causa dor isquêmica, seguida por dano reversível e, eventualmente, irreversível nos tecidos dentro do compartimento. O edema resultante cria um ciclo vicioso, agravando ainda mais a lesão isquêmica. 1

A SC pode se manifestar como aguda, caracterizada por sintomas graves em curto período, ou crônica, de longa duração. A síndrome compartimental aguda (SCA) é uma emergência médica e é tipicamente causada por uma lesão grave ou trauma, resultando em dor intensa. Por outro lado, a SC crônica (SCC) geralmente não é uma emergência médica imediata e costuma surgir após atividade atlética intensa e repetitiva, na ausência de trauma agudo, podendo ser aliviada com descanso e recuperação. 4 5

Junto com outras condições relacionadas ao esforço, a SCC pode contribuir significativamente para o desenvolvimento de sintomas dependentes do esforço. Portanto, essa condição é particularmente importante nos contextos esportivo e do exercício físico. Uma das primeiras descrições de SCC ocorreu durante a expedição britânica ao Polo Sul em 1912, na qual Edward Wilson descreveu o inchaço e a dor no compartimento anterior da perna durante longas caminhadas na região Antártica. Registros históricos subsequentes também enfatizaram a prevalência da SCC em coortes militares, recebendo o apelido de “gangrena de marcha”. 4

## Fisiopatologia

Inúmeras hipóteses foram aventadas para elucidar a fisiopatologia da SCC. No entanto, a verdadeira etiologia e processo de desenvolvimento da SCC permanecem indeterminados. 6

O que se sabe é que o exercício intenso pode levar a um aumento substancial no volume muscular, resultante das demandas metabólicas aumentadas, perfusão tecidual e crescimento das fibras musculares. Quando a pressão dentro do compartimento fascial excede a pressão diastólica, pode ocorrer a síndrome compartimental. Esse fluxo sanguíneo comprometido causa isquemia tecidual, acumulação de metabólitos e dor na área afetada. 4

Alguns pesquisadores sugerem que a SCC não tratada pode levar à compressão neural e danos irreversíveis devido ao vazamento de fluido e aumento das pressões intracompartimentais. A diminuição da densidade capilar e o fluxo venoso prejudicado também foram implicados no desenvolvimento da SCC. 4

A SCC surge devido à elevação da pressão intracompartimental, que pode ser desencadeada por diversos eventos precipitantes. A pressão dentro de um compartimento muscular é influenciada pelo fluxo sanguíneo local, determinado pela pressão arterial local, pressão venosa e resistência vascular. A isquemia ocorre quando a pressão intersticial ultrapassa a pressão de perfusão capilar (PPC). A isquemia do músculo esquelético libera uma substância semelhante à histamina, que aumenta a permeabilidade vascular, causando a formação de trombos sanguíneos e piorando as condições isquêmicas. Miócitos que se rompem liberam proteínas que causam a saída de água do sangue arterial para o compartimento. 7

Outros fatores associados à SCC envolvem métodos de treinamento inadequados, desalinhamento dos membros, discrepâncias no comprimento das pernas, estilo de corrida e controle neuromuscular deficiente. 8

Embora o mecanismo subjacente preciso ainda seja objeto de debate, o consenso aponta para a repetição de esforço muscular dentro de um compartimento, levando a uma redução da perfusão sanguínea como a principal via para o desenvolvimento dos sintomas da SCC. 8

## Epidemiologia e Fatores de Risco

A prevalência real da SCC permanece incerta devido a fatores como: indivíduos que fazem auto tratamento ou modificam suas atividades, erros no diagnóstico clínico e a não busca de atendimento médico. É estimado que de 14 a 34% dos casos de dor nas pernas encaminhadas para tratamento ortopédico relacionadas à atividade ou esforço sejam compatíveis com SCC. 4 A prevalência atual da SCC na população em geral é desconhecida. No entanto, ela foi documentada em subgrupos atléticos específicos com uma taxa de 0,49 casos por mil pessoas por ano. 9

A SCC afeta principalmente a perna, com mais de 95% dos casos relatados nesta região. No entanto, existem relatos variáveis de envolvimento no compartimento paravertebral lombar, 1 na mão, no antebraço, na coxa e no pé em grupos de alto risco específicos. 4 Entre os compartimentos das pernas, o compartimento anterior é o mais comumente afetado (42–60%), seguido pelo lateral (35–36%), posterior profundo (19–32%) e compartimentos superficiais posteriores (3–21%). O envolvimento de um único compartimento é menos comum (37%), com aproximadamente 40% dos casos sintomáticos em 2 compartimentos, 18% envolvendo 3 compartimentos e apenas 5% afetando todos os 4 compartimentos. O envolvimento bilateral é mais prevalente, representando até 95% dos casos, sem diferenças observadas com base na lateralidade. 4

Devido a disparidades anatômicas entre adolescentes e adultos, pacientes mais jovens enfrentam um risco elevado de desenvolver síndrome compartimental. 7 Shadgan et al. 10 observaram que os médicos frequentemente acreditam que os indivíduos mais jovens possuem uma fáscia mais rígida/estreita e forte, combinada com maior densidade muscular, o que aumenta sua vulnerabilidade à síndrome compartimental. Portanto, a SCC é frequentemente diagnosticada, embora não limitada, em jovens desportistas envolvidos em atividades repetitivas, como corridas de longa distância ou *cross-country* . 7

Estudos mais antigos mostravam resultados conflitantes em relação à prevalência da SCC em homens e mulheres. Alguns estudos encontraram uma ocorrência maior em homens, enquanto outros sugeriram um aumento potencial da ocorrência em mulheres. No entanto, as últimas investigações da literatura relataram uma incidência aproximadamente igual de SCC entre homens e mulheres. 4

Rothman et al. 11 descobriram que as mulheres tinham menos probabilidade do que os homens de retornar ao esporte após uma intervenção cirúrgica, e fatores como pressões intracompartimentais, participação esportiva e resultados pós-cirúrgicos não foram estatisticamente diferentes entre os gêneros.

Mais de 90% das pessoas diagnosticadas com SCC estão envolvidas em atividades atléticas, sem diferença relatada entre aqueles que participam de níveis de competição de elite ou recreativas. Embora vários esportes tenham sido associados ao desenvolvimento da SCC, incluindo lacrosse, futebol, basquete, esqui e hóquei de campo, ela é mais comumente observada em corredores de longa distância, representando até 68% dos casos. 4 Há alguns casos raros relatados em atletas envolvidos em levantamento de peso, futebol americano e beisebol. 2 3 12 No entanto, a SCC também pode ocorrer em populações menos ativas.

O exercício intenso, particularmente a corrida, embora não exclusivamente, foi associado a um aumento na incidência de SCC. Mudanças fisiológicas e metabólicas resultantes de atividade física significativa afetam o volume muscular e as pressões compartimentais. O fortalecimento muscular excêntrico em adultos foi identificado como uma possível causa para a diminuição da conformidade fascial e o desenvolvimento da SCC. Pacientes com SCC frequentemente têm uma fáscia espessada e uma maior prevalência de defeitos fasciais em comparação com indivíduos assintomáticos. Esteroides anabólicos androgênicos e outras drogas para melhora de desempenho que são usadas para o aumento muscular também podem contribuir para elevações anormais nas pressões intracompartimentais e foram sugeridos como possíveis fatores de risco para a SCC. 4 Alguns indícios sugerem que erros de treinamento, especificamente um aumento abrupto no volume e/ou intensidade do treinamento, podem ser o fator de risco mais importante para o desenvolvimento da SCC. 13

## Anatomia e Apresentação Clínica

A SCC é a segunda causa mais comum de dor nas pernas relacionada ao esforço, seguida da síndrome do estresse tibial medial (SETM), afetando aproximadamente um terço dos atletas. É importante considerar outros potenciais diagnósticos de dor nas pernas induzida pelo exercício, como compressão nervosa, lesões ósseas por estresse, trombose venosa profunda, SETM e outras condições clínicas ao avaliar os sintomas. 4 13

Um conhecimento abrangente da anatomia dos membros inferiores é crucial para diagnosticar a SCC e identificar os compartimentos envolvidos. A perna inferior é dividida anatomicamente em quatro compartimentos: anterior, lateral, superficial posterior e posterior profundo, com um quinto compartimento adicional para o músculo tibial posterior, que possui sua própria fáscia ( Fig. 1 ). 14

**Fig. 1:** Corte transversal da perna, evidenciando os quatro compartimentos: o compartimento anterior, onde se alocam os músculos tibial anterior, extensores longos dos dedos dos pés, extensor longo do hálux e fibular terceiro, o compartimento lateral composto pelos músculos fibular longo e curto, o compartimento superficial posterior composto pelos músculos gastrocnêmio, sóleo e plantares e o compartimento posterior profundo contendo o músculo tibial posterior e os flexores longos dos dedos dos pés.

O compartimento anterior abriga o nervo fibular profundo, artéria tibial anterior, músculo tibial anterior, extensores longos dos dedos dos pés, extensor longo do hálux e fibular terceiro. A pressão aumentada neste compartimento pode levar à perda sensorial no primeiro espaço interdigital e fraqueza na dorsiflexão dos dedos dos pés e tornozelo. 14

O compartimento lateral é composto pelos músculos fibular longo e curto, artéria fibular, bem como pelo nervo fibular superficial. A compressão neste compartimento pode causar fraqueza na eversão do pé e redução da sensibilidade no dorso do pé. 14

O compartimento superficial posterior contém a artéria tibial posterior, os músculos gastrocnêmio, sóleo, plantares, juntamente com o segmento distal do nervo sural. A compressão aqui pode resultar em dormência na lateral do pé e na panturrilha distal. 14

O compartimento posterior profundo contém o músculo tibial posterior, flexores longos dos dedos dos pés, a artéria fibular e o nervo tibial. O aumento da pressão neste compartimento pode levar a fraqueza na flexão plantar e dormência na planta do pé. 14 Dado que a grande maioria dos casos ocorre nos membros inferiores, não entraremos em detalhes anatômicos de outras regiões.

A progressão natural da SCC geralmente é atraumática, embora alguns indivíduos possam relatar uma história de trauma de baixa energia. Os pacientes frequentemente experimentam sensação de rigidez, dor ou desconforto nas partes anterior e lateral da perna após exercício prolongado, com sintomas que geralmente melhoram com repouso ou redução da atividade, sendo esse detalhe, em particular, considerado um critério específico da doença. 13 Os sintomas da SCC costumam ser bilaterais em até 95% dos pacientes. Junto com a distribuição do nervo fibular superficial, os indivíduos afetados também podem apresentar redução da sensibilidade vibratória e amplitude motora reduzida, levando a um menor controle do pé e tornozelo, como descrito acima. 4 Cãibras, hipossensibilidade ou fraqueza muscular são evidentes em aproximadamente um terço dos pacientes. 13

## Diagnóstico

O diagnóstico da SCC se baseia em um histórico detalhado e exame físico minucioso. É importante documentar a frequência, volume, duração e intensidade das sessões de treinamento, bem como quaisquer padrões no início e na resolução dos sintomas relatados. Embora os pacientes possam não apresentar sintomas em repouso, o esforço pode desencadear sintomas significativos que limitam a atividade. Os cinco sintomas principais frequentemente relatados por pacientes com SCC incluem: dor, aperto, cãibras, fraqueza e diminuição da sensibilidade no dorso do pé. 4

Recentemente, Vogels et al. 15 propuseram cinco critérios-chave para o diagnóstico da SCC. Os membros do painel do estudo concordaram que a SCC era considerada provável se o paciente (I) estiver envolvido em atividades que exigem ativação repetitiva dos mesmos músculos, (II) relatar dor durante o exercício, (III) relatar rigidez/aperto durante o exercício, (IV) interromper precocemente ou evitar atividades específicas e (V) se ele pode induzir sintomas realizando atividades provocativas durante o exame físico. 15

Durante o exame físico, a dor pode ser provocada pelo alongamento passivo de um compartimento se o paciente tiver se exercitado recentemente, embora isso seja incomum em repouso. A palpação da área afetada pode revelar defeitos na fáscia, encontrados em 39 a 46% das pessoas com SCC. 4

Embora haja muito debate em curso, o padrão histórico para o diagnóstico da SCC tem sido a medição da pressão intracompartimental. Os pioneiros no desenvolvimento de uma técnica com tal objetivo foram Whitesides et al., 16 em um estudo revolucionário no qual utilizaram objetos como seringa, agulha, solução salina e tubos de plástico conectados a um manômetro. Atualmente, o monitoramento da pressão intracompartimental pode ser realizado usando diversos dispositivos comercialmente disponíveis, os quais foram inspirados pela invenção de Whitesides et al. 16 Isso permite a comparação dos compartimentos afetados e não afetados nos membros inferiores bilaterais. No processo diagnóstico, os pacientes realizam um teste de estresse físico. Uma série de medições de manometria é feita antes e após o exercício para analisar as tendências das pressões intracompartimentais nos compartimentos sintomáticos. A pressão intracompartimental típica em repouso na perna costuma ser menor que 10 mmHg, embora as medidas possam variar consideravelmente entre os pacientes e sejam influenciadas pelo operador que realiza a medição. 4

Em um estudo conduzido por Davis et al., 17 pacientes com SCC foram monitorados durante testes de estresse físico. A análise revelou que esses indivíduos experimentavam dor na perna após uma média de 11 minutos de esforço, classificando a dor como 8 de 10 em na Escala Visual Analógica. Os sintomas diminuíam após cerca de 45 minutos de repouso. Aproximadamente 36% dos pacientes também relataram dormência ou formigamento além da dor após o esforço. Medidas objetivas de pressão nos compartimentos mostraram aumentos significativos nos compartimentos anterior, lateral, posterior profundo e superficial posterior após os testes de estresse físico. 4

Pedowitz et al. 18 estabeleceram critérios diagnósticos para confirmar a presença de SCC induzida por exercícios. Segundo eles, o diagnóstico pode ser feito se um dos seguintes critérios for atendido: 1) pressão pré-exercício maior que 15 mmHg; 2) pressão 1 minuto após o exercício maior que 30 mmHg; ou 3) pressão 5 minutos após o exercício maior que 20 mmHg.

Contudo, vale destacar que os critérios de corte diagnóstico diferem substancialmente de acordo com o autor, 15 e as medições de pressão nem sempre são confiáveis devido a fatores como tolerância do paciente, técnica do operador e uso de diferentes dispositivos de medição. Além disso, a natureza invasiva do teste pode estar associada a riscos de colocação incorreta da agulha, formação de hematomas e dano nervoso. 15

Aweid et al. 4 revisaram diversos estudos avaliando a utilidade das medições de pressão intracompartimental no diagnóstico da SCC. Eles concluíram que, embora as medições de pressão sejam comumente utilizadas, há evidências limitadas para validar sua precisão, e a apresentação clínica deve ser mais considerada no diagnóstico da SCC.

Dado o questionamento quanto à aplicabilidade e valor diagnóstico da aferição de pressão intracompartimental, é essencial ter em mente que a história clínica, o exame físico e a exclusão de diagnósticos diferenciais são componentes indispensáveis do processo diagnóstico. Com o intuito de superar essas limitações da técnica da agulha, novos protocolos diagnósticos foram sugeridos, sendo recomendado até mesmo o uso sistemático de ressonância magnética (RM) convencional para a exclusão de diagnósticos diferenciais. 19

A RM é o melhor método de imagem para avaliar a dor na perna relacionada ao exercício, pois detecta condições como síndrome do estresse tibial, fratura por estresse tibial, compressões neurais, lesões musculares e tendinosas, trombose relacionada ao exercício e hérnias fasciais. 19

Na prática clínica, primeiramente, sequências de RM convencional são realizadas para excluir diagnósticos diferenciais. Posteriormente, os pacientes são submetidos à corrida (ou até mesmo caminhada) em esteira, de acordo com suas capacidades físicas, até que não possam mais tolerar a atividade devido à dor. Logo após interromperem a atividade, retornam imediatamente à RM para a aquisição de sequências axiais sensíveis a líquidos e suprimidas de gordura (ponderadas em T2/ *short tau inversion recovery* , STIR). Alguns estudos confirmaram a validade da RM pós-exercício no diagnóstico da SCC, utilizando um aumento de 1,54 vezes na intensidade do sinal como valor de corte diagnóstico, com sensibilidade de 96% e especificidade entre 87 e 90%. 19

Portanto, a RM é um método não invasivo, prontamente aceito pelos pacientes, com boa disponibilidade em grandes centros médicos, sendo o melhor exame para a exclusão de diagnósticos diferenciais e uma opção cientificamente validada para o diagnóstico da SCC. 19

## Tratamento

## Tratamento Cirúrgico

Diversas estratégias de manejo, operatórias e não operatórias, foram descritas para o tratamento da SCC. Tradicionalmente, o tratamento cirúrgico apresenta mais descrições e melhores resultados, embora haja evidências crescentes de que o tratamento conservador pode ser uma opção em determinados casos. Em atletas, o manejo consiste em intervenção cirúrgica. A falha no tratamento não cirúrgico, sensação de parestesia, dor ao esforço que desaparece com o repouso, aperto, cãibra, isquemia, pé caído e desejo do paciente são as principais indicações para o tratamento cirúrgico. 9 20

Em uma revisão sistemática sobre manejo cirúrgico da SCC, 9 que incluiu 1.495 pacientes de 24 estudos, as técnicas mais utilizadas foram: fasciotomia aberta específica para o compartimento (86%), fasciotomia com fasciectomia parcial (12%) e fasciotomia endoscópica (< 2%). Para o compartimento anterior, o mais comumente afetado, uma única incisão longitudinal entre a crista tibial anterior e a fíbula através da pele e tecido subcutâneo foi a mais frequente (207 de 240). No entanto, os resultados da revisão não demonstraram se havia superioridade entre as técnicas descritas. Em relação ao compartimento posterior, a técnica cirúrgica mais utilizada foi uma incisão longitudinal ligeiramente medial à crista tibial com liberação da ponte solar da tíbia para acessar a fáscia profunda. A taxa de sucesso desta intervenção foi de 61% para o compartimento posterior profundo (44 de 72) e 100% para o posterior superficial (3 de 3), os autores sugerem que este compartimento tem sido propenso a um menor sucesso cirúrgico.

Uma revisão sistemática, a qual incluiu sete artigos, sobre intervenção cirúrgica no compartimento posterior constatou que as técnicas diferiram ligeiramente ao longo dos estudos, sem obter uma conclusão final, uma vez que os pesquisadores utilizaram diferentes métodos para mensuração dos resultados. 21

Novos procedimentos, como os minimamente invasivos ou os endoscópicos, vêm ganhando espaço nas últimas décadas. Uma revisão de Lohrer et al. 13 concluiu que não havia diferença estatística entre essas técnicas: a taxa de sucesso média não ponderada foi de 86,3% para a técnica endoscópica e 80,0% para liberação minimamente invasiva de SCC. D'Amore et al. 22 conduziram um estudo comparando o procedimento endoscópico e a fasciotomia aberta em atletas, de elite e amadores, com SCC dos membros inferiores. Os resultados mostraram que a taxa de retorno ao esporte foi de 84,6% naqueles submetidos a procedimento endoscópico e 72,7% naqueles submetidos a fasciotomia aberta, e a recorrência dos sintomas foi de 69,2% e 72,7%, respectivamente, sem diferença estatística. Nenhuma complicação ou resultado grave foi descrito em nenhum dos grupos.

A técnica endoscópica teria benefícios sobre a fasciotomia aberta, como menor risco de infecção, menor tempo de retorno mais rápido à atividade, devido à menor manipulação dos tecidos moles, menor hematoma pós-operatório, fibrose limitada, melhor visualização das estruturas dentro do compartimento e extensão da liberação fascial. 9 22 Como tratamento alternativo para a liberação da SCC dos compartimentos anterior e lateral, tem se mostrado um tratamento adequado, com boa taxa de sucesso, sem inferioridade na literatura. 22

## Tratamento Conservador

O tratamento conservador da SCC induzida por exercícios mantém-se pouco documentado na pesquisa científica. Repouso, cessação da atividade que desencadeia os sintomas e analgésicos parecem ser os fundamentos básicos; contudo, existem poucas diretrizes documentadas ou procedimentos específicos de como otimizá-los e para qual população seriam mais adequados. Em uma revisão sistemática da literatura sobre novos manejos não cirúrgicos, Rajasekaran et al. 6 encontraram poucas evidências sobre as técnicas apresentadas, as quais incluíam mudança de marcha, quimiodenervação, fenestração da fáscia guiada por ultrassom e massagem.

No entanto, a série de estudos de caso feitos por Diebal et al., 23 com observação de diferentes técnicas de corrida e como elas afetam a pressão do compartimento e a dor dos pacientes com SCC, mostraram-se promissoras e foram incluídas em um programa militar de manejo não cirúrgico. 23 24 Esse protocolo envolve diversos tratamentos descritos na literatura, tendo um foco maior principalmente na reeducação da marcha e da corrida. Em um de seus estudos, eles verificaram que, após um acompanhamento de 2 anos em uma população de 50 pacientes submetidos a esse protocolo, 57% ainda estavam na ativa sem cirurgia, 43% retornaram ao posto militar original, 36% deixaram o serviço militar, 48% ainda apresentavam sintomas e 12% dos pacientes receberam fasciotomia. 24 Este estudo mostra resultados moderados, que poderiam levar à diminuição na necessidade de procedimentos cirúrgicos. Há poucas evidências sobre retreinamento da marcha na população atlética, no entanto, esses focos de pesquisa podem ser uma alternativa a fasciotomia ou uma tentativa de preveni-la.

Em 2022, um painel de consenso clínico composto por especialistas discutiu o tratamento conservador da SCC e qual poderia ser mais eficaz, chegando à conclusão de que o retreinamento da marcha e a cessação das atividades provocativas são de grande importância quando se tenta uma abordagem não cirúrgica. A literatura relata que o tratamento por fisioterapeutas, injeções botulínicas ou modificações de calçados são de menor importância e principalmente medidas adjacentes a serem utilizadas dependendo dos sintomas do paciente. 15

## Tratamento Conservador *Versus* Cirúrgico

Embora não existam ensaios clínicos com pacientes randomizados para comparar o tratamento cirúrgico com o conservador, alguns estudos têm encontrado superioridade nos procedimentos intervencionistas, principalmente em pacientes com SCC do compartimento anterior e em atletas, tanto de nível amador quanto de elite. Em uma coorte retrospectiva, Vogels et al. 25 constataram que a taxa de sucesso considerada pelos pacientes foi significativamente maior naqueles submetidos à fasciotomia (42%, em comparação com 17% de taxa de sucesso no grupo que recebeu tratamento conservador), e também foi notada uma menor frequência de dor e aperto durante a prática de esportes. Contudo, não houve diferença entre esses dois grupos quando se tratava de retornar ao mesmo nível de desempenho que tinham antes de cada intervenção.

Em contraste com esses achados, Thein et al. 26 observaram resultados significativamente melhores no retorno ao esporte e manutenção do mesmo nível de atividade física, medido pelo escore de Tegner, naqueles submetidos ao tratamento cirúrgico. A taxa de retorno ao nível atlético pré-sintomático foi de 25% nos pacientes que não realizaram fasciotomia e 77,4% para os que o fizeram, com *p* = 0,001.

É importante ressaltar que nenhum desses estudos possui o padrão-ouro quanto ao procedimento metodológico, existem vários vieses inerentes à forma como foram conduzidos e que nenhum deles padronizou o tratamento conservador, o qual foi realizado em sua maioria por fisioterapeutas ou clínicos. Porém, corroboram com a hipótese de que para atletas, o procedimento cirúrgico parece ser superior como tratamento e para a satisfação do paciente. 25 26

## Considerações Finais

A SCC é a segunda causa mais comum de dor após esforço em membros inferiores, seguida da síndrome do estresse tibial medial. Apesar de raramente ser urgente e os sintomas serem aliviados pelo repouso e pelo cessamento das atividades desencadeantes, a avaliação precisa da pressão intracompartimental se mantém desafiadora e o tratamento padrão-ouro impreciso. Ademais, alguns autores sugerem que a SCC não tratada pode levar à compressão neural e danos irreversíveis devido às pressões elevadas intracompartimental.

A prevalência real da SCC permanece incerta, mas estima-se que seja responsável por aproximadamente 14 a 34% das dores nas pernas relacionadas à atividade física. Exercícios intensos, particularmente a corrida, foram relacionados a um aumento na incidência de SCC; entretanto, ela também é comumente reportada em militares.
